# Well-being is more than happiness and life satisfaction: a multidimensional analysis of 21 countries

**DOI:** 10.1186/s12955-020-01423-y

**Published:** 2020-06-19

**Authors:** Kai Ruggeri, Eduardo Garcia-Garzon, Áine Maguire, Sandra Matz, Felicia A. Huppert

**Affiliations:** 1 Columbia University Mailman School of Public Health, New York, USA; 2 Policy Research Group, Centre for Business Research, Judge Business School, University of Cambridge, Cambridge, UK; 3 Universidad Camilo José Cela, Madrid, Spain; 4 Trinity College Dublin, Dublin, Ireland; 5 Columbia Business School, New York, USA; 6 Australia Catholic University, Sydney, Australia; 7 Well-being Institute, University of Cambridge, Cambridge, UK

**Keywords:** Well-being, Mental health, Composite measures, Economic policy, Exploratory structural equation models

## Abstract

**Background:**

Recent trends on measurement of well-being have elevated the scientific standards and rigor associated with approaches for national and international comparisons of well-being. One major theme in this has been the shift toward multidimensional approaches over reliance on traditional metrics such as single measures (e.g. happiness, life satisfaction) or economic proxies (e.g. GDP).

**Methods:**

To produce a cohesive, multidimensional measure of well-being useful for providing meaningful insights for policy, we use data from 2006 and 2012 from the European Social Survey (ESS) to analyze well-being for 21 countries, involving approximately 40,000 individuals for each year. We refer collectively to the items used in the survey as multidimensional psychological well-being (MPWB).

**Results:**

The ten dimensions assessed are used to compute a single value standardized to the population, which supports broad assessment and comparison. It also increases the possibility of exploring individual dimensions of well-being useful for targeting interventions. Insights demonstrate what may be masked when limiting to single dimensions, which can create a failure to identify levers for policy interventions.

**Conclusions:**

We conclude that both the composite score and individual dimensions from this approach constitute valuable levels of analyses for exploring appropriate policies to protect and improve well-being.

## Background

### What is well-being?

Well-being has been defined as the combination of feeling good and functioning well; the experience of positive emotions such as happiness and contentment as well as the development of one’s potential, having some control over one’s life, having a sense of purpose, and experiencing positive relationships [23]. It is a sustainable condition that allows the individual or population to develop and thrive. The term subjective well-being is synonymous with positive mental health. The World Health Organization [45] defines positive mental health as “a state of well-being in which the individual realizes his or her own abilities, can cope with the normal stresses of life, can work productively and fruitfully, and is able to make a contribution to his or her community”. This conceptualization of well-being goes beyond the absence of mental ill health, encompassing the perception that life is going well.

Well-being has been linked to success at professional, personal, and interpersonal levels, with those individuals high in well-being exhibiting greater productivity in the workplace, more effective learning, increased creativity, more prosocial behaviors, and positive relationships [10, 27, 37]. Further, longitudinal data indicates that well-being in childhood goes on to predict future well-being in adulthood [39]. Higher well-being is linked to a number of better outcomes regarding physical health and longevity [13] as well as better individual performance at work [30], and higher life satisfaction has been linked to better national economic performance [9].

### Measurement of well-being

Governments and researchers have attempted to assess the well-being of populations for centuries [2]. Often in economic or political research, this has ended up being assessed using a single item about life satisfaction or happiness, or a limited set of items regarding quality of life [3]. Yet, well-being is a multidimensional construct, and cannot be adequately assessed in this manner [14, 24, 29]. Well-being goes beyond hedonism and the pursuit of happiness or pleasurable experience, and beyond a global evaluation (life satisfaction): it encompasses how well people are functioning, known as eudaimonic, or psychological well-being. Assessing well-being using a single subjective item approach fails to offer any insight into how people experience the aspects of their life that are fundamental to critical outcomes. An informative measure of well-being must encompass all the major components of well-being, both hedonic and eudaimonic aspects [2], and cannot be simplified to a unitary item of income, life satisfaction, or happiness.

Following acknowledgement that well-being measurement is inconsistent across studies, with myriad conceptual approaches applied [12], Huppert and So [27] attempted to take a systematic approach to comprehensively measure well-being. They proposed that positive mental health or well-being can be viewed as the complete opposite to mental ill health, and therefore attempted to define mental well-being in terms of the opposite of the symptoms of common mental disorders. Using the DSM-IV and ICD-10 symptom criteria for both anxiety and depression, ten features of psychological well-being were identified from defining the opposite of common symptoms. The features encompassed both hedonic and eudaimonic aspects of well-being: competence, emotional stability, engagement, meaning, optimism, positive emotion, positive relationships, resilience, self-esteem, and vitality. From these ten features an operational definition of flourishing, or high well-being, was developed using data from Round 3 of the European Social Survey (ESS), carried out in 2006. The items used in the Huppert and So [27] study were unique to that survey, which reflects a well-being framework based on 10 dimensions of good mental health. An extensive discussion on the development and validation of these measures for the framework is provided in this initial paper [27].

As this was part of a major, multinational social survey, each dimension was measured using a single item. As such, ‘multidimensional’ in this case refers to using available measures identified for well-being, but does not imply a fully robust measure of these individual dimensions, which would require substantially more items that may not be feasible for population-based work related to policy development. More detailed and nuanced approaches might help to better capture well-being as a multidimensional construct, and also may consider other dimensions. However, brief core measures such as the one implemented in the ESS are valuable as they provide a pragmatic way of generating pioneering empirical evidence on well-being across different populations, and help direct policies as well as the development of more nuanced instruments. While this naturally would benefit from complementary studies of robust measurement focused on a single topic, appropriate methods for using sprawling social surveys remain critical, particularly through better standardization [6]. While this paper will overview those findings, we strongly encourage more work to that end, particularly in more expansive measures to support policy considerations.

### General approach and key questions

The aim of the present study was to develop a more robust measurement of well-being that allows researchers and policymakers to measure well-being both as a composite construct and at the level of its fundamental dimensions. Such a measure makes it possible to study overall well-being in a manner that goes beyond traditional single-item measures, which capture only a fraction of the dimensions of well-being, and because it allows analysts to unpack the measure into its core components to identify strengths and weaknesses. This would produce a similar approach as the most common reference for policy impacts: Gross Domestic Product (GDP), which is a composite measure of a large number of underlying dimensions.

The paper is structured as follows: in the first step, data from the ESS are used to develop a composite measure of well-being from the items suggested by Huppert & So [27] using factor analysis. In the second step, the value of the revised measure is demonstrated by generating insights into the well-being of 21 European countries, both at the level of overall well-being and at the level of individual dimensions.

## Methods

### The European social survey

The ESS is a biannual survey of European countries. Through comprehensive measurement and random sampling techniques, the ESS provides a representative sample of the European population for persons aged 15 and over [38]. Both Round 3 (2006–2007) and Round 6 (2012–2013) contained a supplementary well-being module. This module included over 50 items related to all aspects of well-being including psychological, social, and community well-being, as well as incorporating a brief measure of symptoms of psychological distress. As summarized by Huppert et al. [25], of the 50, only 30 items relate to personal well-being, of which only 22 are positive measures. Of those remaining, not all relate to the 10 constructs identified by Huppert and So [27], so only a single item could be used, or else the item that had the strongest face validity and distributional items were chosen.

Twenty-two countries participated in the well-being modules in both Round 3 and Round 6. As this it within a wider body of analyses, it was important to focus on those initially. Hungary did not have data for the vitality item in Round 3 and was excluded from the analysis, as appropriate models would not have been able to reliably resolve a missing item for an entire country. To be included in the analysis and remain consistent, participants therefore had to complete all 10 items used and have the age, gender, employment, and education variables completed. Employment was classified into four groups: students, employed, unemployed, retired; other groups were excluded. Education was classified into three groups: low (less than secondary school), middle (completed secondary school), and high (postsecondary study including any university and above). Using these criteria, the total sample for Round 6 was 41,825 people from 21 countries for analysis. The full sample was 52.6% female and ranged in age from 15 to 103 (M = 47.9; SD = 18.9). Other details about participation, response rates, and exclusion have been published elsewhere [38].

### Measurement of well-being

Huppert & So [27] defined well-being using 10 items extracted from the Round 3 items, which represent 10 dimensions of well-being. However, the items used in Round 3 to represent positive relationships and engagement exhibited ceiling effects and were removed from the questionnaire in Round 6. Four alternatives were available to replace each question. Based on their psychometric properties (i.e., absence of floor effects and wider response distributions), two new items were chosen for positive relationships and engagement (one item for each dimension). The new items and those they replaced can be seen in Table 1 (also see Supplement).

**Table 1:**
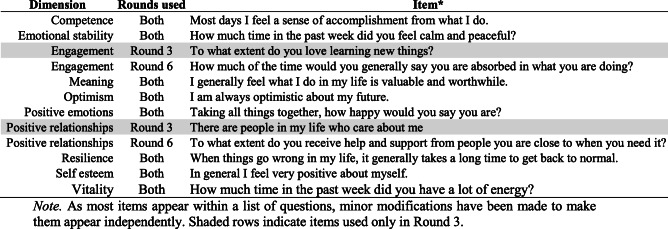
ESS items by dimension used in Round 3 and Round 6

### Development of a composite measure of psychological well-being (MPWB)

A composite measure of well-being that yields an overall score for each individual was developed. From the ten indicators of well-being shown in Table 1, a single factor score was calculated to represent MPWB. This overall MPWB score hence constitutes a summary of how an individual performs across the ten dimensions, which is akin to a summary score such as GDP, and will be of general value to policymakers. Statistical analysis was performed in R software, using lavaan [40] and lavaan.survey [35] packages. The former is a widely-used package for the R software designed for computing structural equation models and confirmatory factor analyses (CFA). The latter allows introducing complex survey design weights (combination of design and population size weights) when estimating confirmatory factor analysis models with lavaan, which ensures that MPWB scoring followed ESS guidelines regarding both country-level and survey specific weights [17]. Both packages have been previously tested and validated in various analyses using ESS data (as explained in detail in lavaan.survey documentation).

It should be noted that Round 6 was treated as the focal point of these efforts before repeating for Round 3, primarily due to the revised items that were problematic in Round 3, and considering that analyses of the 2006 data are already widely available.

Prior to analysis, all items were coded such that higher scores were more positive and lower scores more negative. Several confirmatory factor analysis models were performed in order to test several theoretical conceptualizations regarding MPWB. Finally, factor scores (expected *a posteriori* [15];) were calculated for the full European sample and used for descriptive purposes. The approach and final model are presented in supplemental material.

Factor scores are individual scores computed as weighted combinations of each person’s response on a given item and the factor scoring coefficients. This approach is to be preferred to using raw or sum scores: sum or raw scores fail to consider how well a given item serves as an indicator of the latent variable (i.e., all items are unrealistically assumed to be perfect and equivalent measures of MPWB). They also do not take into account that different items could present different variability, which is expected to occur if items present different scales (as in our case). Therefore, the use of such simple methods results in inaccurate individual rankings for MPWB. To resolve this, factor scores are both more informative and more accurate, as they avoid the propagation of measurement error in subsequent analyses [19].

Not without controversy (see Supplement), factor scores are likely to be preferable to sum scores when ranking individuals on unobservable traits that are expected to be measured with noticeable measurement error (such as MPWB [32];). Similar approaches based on factor scoring have been successfully applied in large international assessment research [21, 34]. With the aim of developing a composite well-being score, it was necessary to provide a meaningful representation of how the different well-being indicators are reflected in the single measure. A hierarchical model with one higher-order factor best approximated MPWB along with two first-order factors (see supplement Figure S1). This model replicates the factor structure reported for Round 3 by Huppert & So [27]. The higher-order factor explained the relationship between two first-order factors (positive functioning and positive characteristics showed a correlation of ρ = .85). In addition, modelling standardized residuals showed that the items representing vitality and emotional stability and items representing optimism and self-esteem were highly correlated. The similarities in wording in both pairs of items (see Table 1) are suspected to be responsible for such high residual correlations. Thus, those correlations were included in the model. As presented in Table 2, the hierarchical model was found to fit the data better than any other model but a bi-factor model including these correlated errors. The latter model resulted in collapsed factor structure with a weak, bi-polar positive functioning factor. However, this bi-factor model showed a problematic bi-polar group factor with weak loadings. Whether this group factor was removed (resulting in a S-1 bi-factor model, as in [16]), model fit deteriorated. Thus, neither bi-factor alternative was considered to be acceptable.

**Table 2:** Fit indices for the different proposed models

Model	χ^2^ (df)	CFI	TLI	RMSEA	SRMR
Single-factor model	4951.62 (35)*	.87	.84	.07 (.07,.07)	.04
Correlated two factors model	3827.68 (34)*	.90	.87	.06 (.06, .06)	.04
Hierarchical model with no correction	3827.68 (34)*	.90	.87	.06 (.06, .06)	.04
Hierarchical model with correction	1541.28 (32)*	.96	.95	.04 (.04, .04)	.02
Bi-factor model	2088.43 (25)*	.95	.91	.05 (.05, .05)	.03
Bi-factor model with correlated errors	1479.327 (23)*	.97	.96	.04 (.04, .04)	.02
S-1 Bi-factor model and correlated errors	2018.361 (27)*	.96	.95	.05 (.04, .05)	.02

*Note =* * significant at 0.01

To calculate the single composite score representing MPWB, a factor scoring approach was used rather than a simplistic summing of raw scores on these items. Factor scores were computed and standardized for the sample population as a whole, which make them suitable for broad comparison [8]. This technique was selected for two reasons. First, it has the ability to take into account the different response scales used for measuring the items included in the multidimensional well-being model. The CFA model, from which MPWB scores were computed, was defined such that the metric of the MPWB was fixed, which results in a standardized scale. Alternative approaches, such as sum or raw scores, would result in ignoring the differential variability across items, and biased individual group scores. Our approach, using factor scoring, resolves this issue by means of standardization of the MPWB scores. The second reason for this technique is that it could take account of how strongly each item loaded onto the MPWB factor. It should be noted that by using only two sub-factors, the weight applied to the general factor is identical within the model for each round. This model was also checked to ensure it also was a good fit for different groups based on gender, age, education and employment.

Separate CFA analyses per each country indicate that the final model fit the data adequately in all countries (.971 < CFI < .995; .960 < TFI < .994; .020 < RMSEA < .05; 0,023 < SRMR < 0,042). All items presented substantive loadings on their respective factors, and structures consistently replicated across all tested countries. Largest variations were found when assessing the residual items’ correlations (e.g., for emotional stability and vitality correlation, values ranged from 0,076 to .394). However, for most cases, residuals correlations were of similar size and direction (for both cases, the standard deviation of estimated correlations was close of .10). Thus, strong evidence supporting our final model was systematically found across all analyzed countries. Full results are provided in the supplement (Tables S2-S3).

#### Model invariance

In order to establish meaningful comparisons across groups within and between each country, a two-stage approach was followed, resulting in a structure that was successfully found to be similar across demographics. First, a descriptive comparison of the parameter estimates unveiled no major differences across groups. Second, factor scores were derived for the sample, employing univariate statistics to compare specific groups within country and round. In these analyses, neither traditional nor modern approaches to factor measurement invariance were appropriate given the large sample and number of comparisons at stake ([8]; further details in Supplement).

From a descriptive standpoint, the hierarchical structure satisfactorily fit both Round 3 and Round 6 data. All indicators in both rounds had substantial factor loadings (i.e., λ > .35). A descriptive comparison of parameter estimates produced no major differences across the two rounds. The lack of meaningful differences in the parameter estimates confirms that this method for computing MPWB can be used in both rounds.

#### Scaling

As MPWB scores from both rounds are obtained from different items that have different scales for responses, it is necessary to transform individual scores obtained from both rounds in order to be aligned. To do this between Round 3 and Round 6 items, a scaling approach was used. To produce common metrics, scores from Round 3 were rescaled using a mean and sigma transformation (Kolen & Brennan 2010) to align with Round 6 scales. This was used as Round 6 measures were deemed to have corrected some deficiencies found in Round 3 items. This does not change outcomes in either round but simply makes the scores match in terms of distributions relative to their scales, making them more suitable for comparison.

## Results

**Fig. 1:**
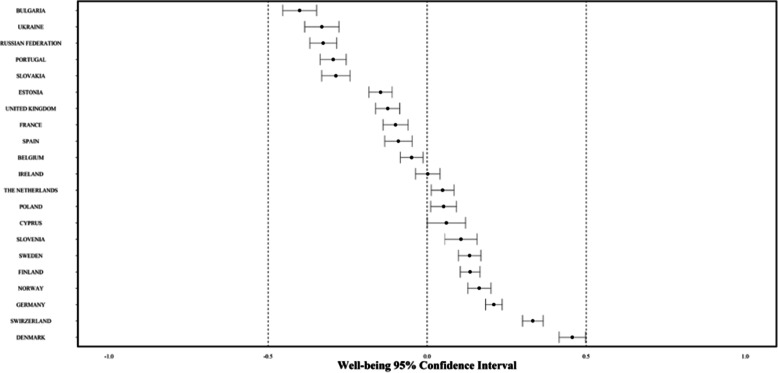
Distribution of national MPWB means and confidence intervals across Europe

As extensive descriptive insights on the sample and general findings are already available (see [41]), we focus this section on the evidence derived directly from the proposed approach to MPWB scores. For the combined single score for MPWB, the overall mean (for all participants combined) is fixed to zero, and the scores represent deviation from the overall mean. In 2012 (Round 6), country scores on well-being ranged from − 0.41 in Bulgaria to 0.46 in Denmark (Fig. 1). There was a significant, positive relationship between national MPWB mean scores and national life satisfaction means (*r =* .56 (.55–.57), *p* < .001). In addition, MPWB was negatively related with depression scores and positively associated with other well-being measurements (see Supplement).

**Fig. 2:**
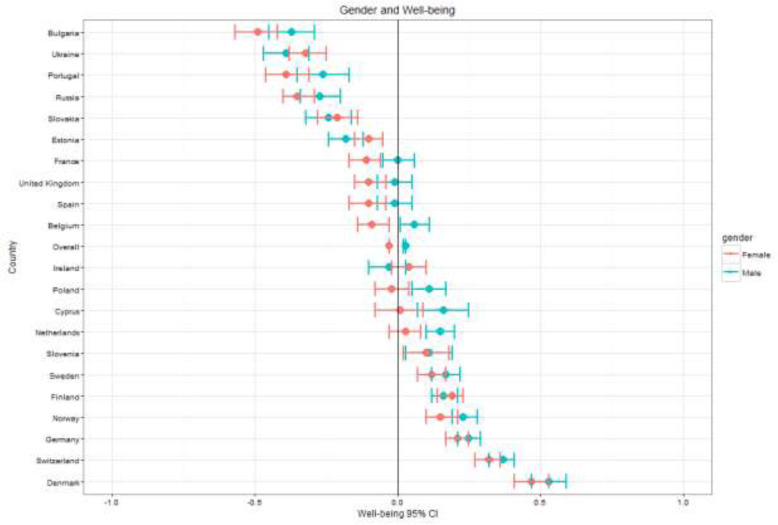
Well-being by country and gender

**Fig. 3:**
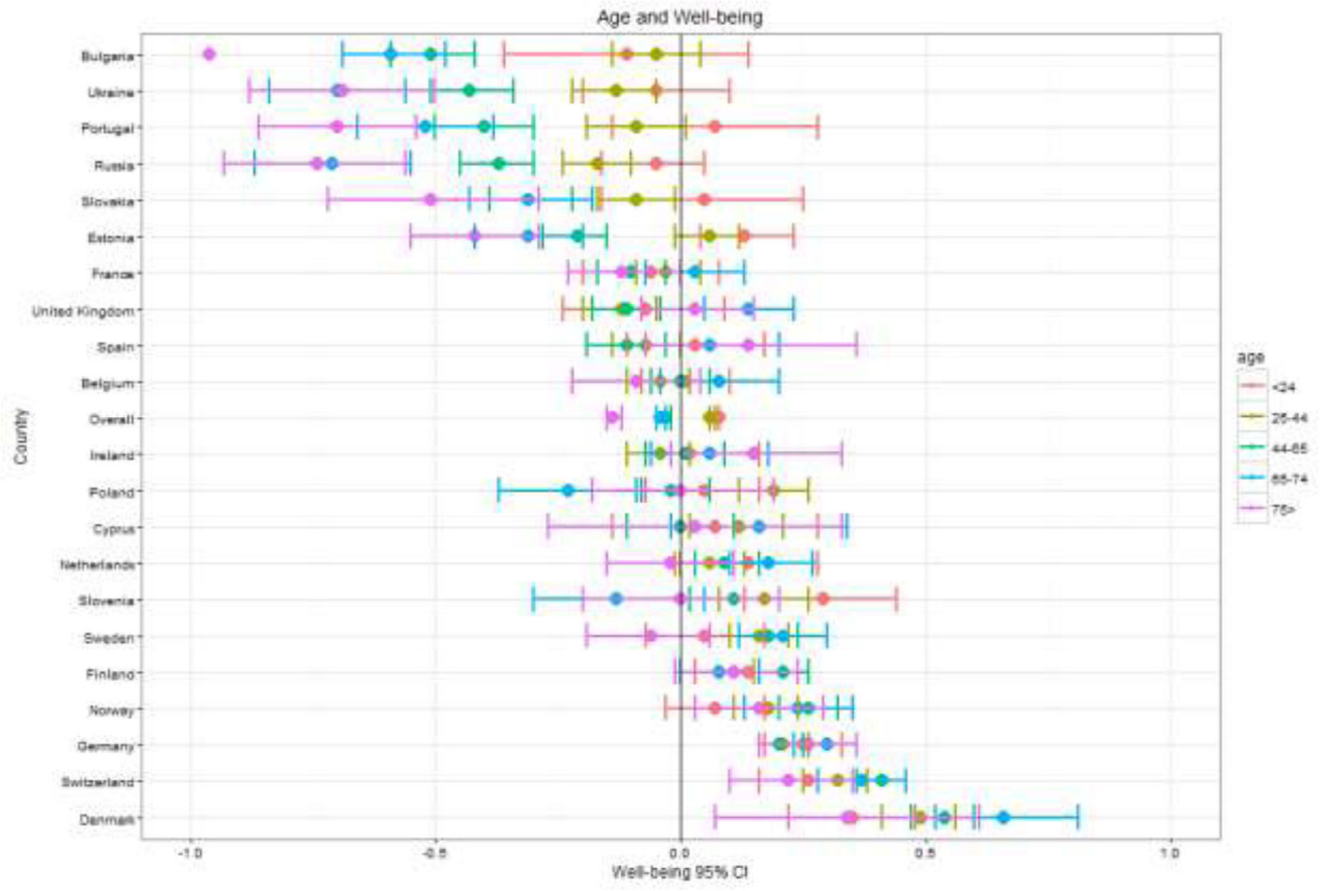
Well-being by country and age

**Fig. 4:**
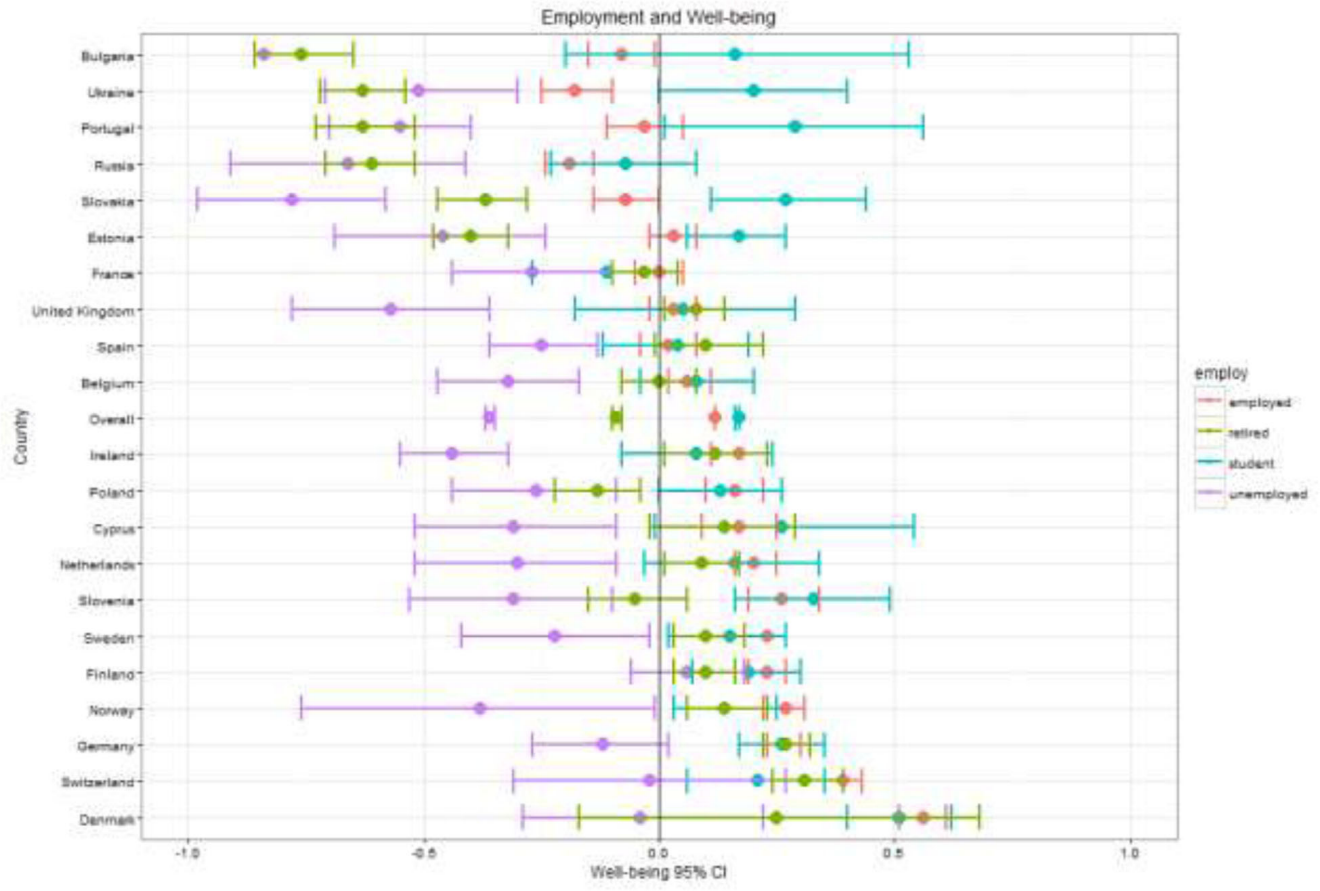
Well-being by country and employment

**Fig. 5:**
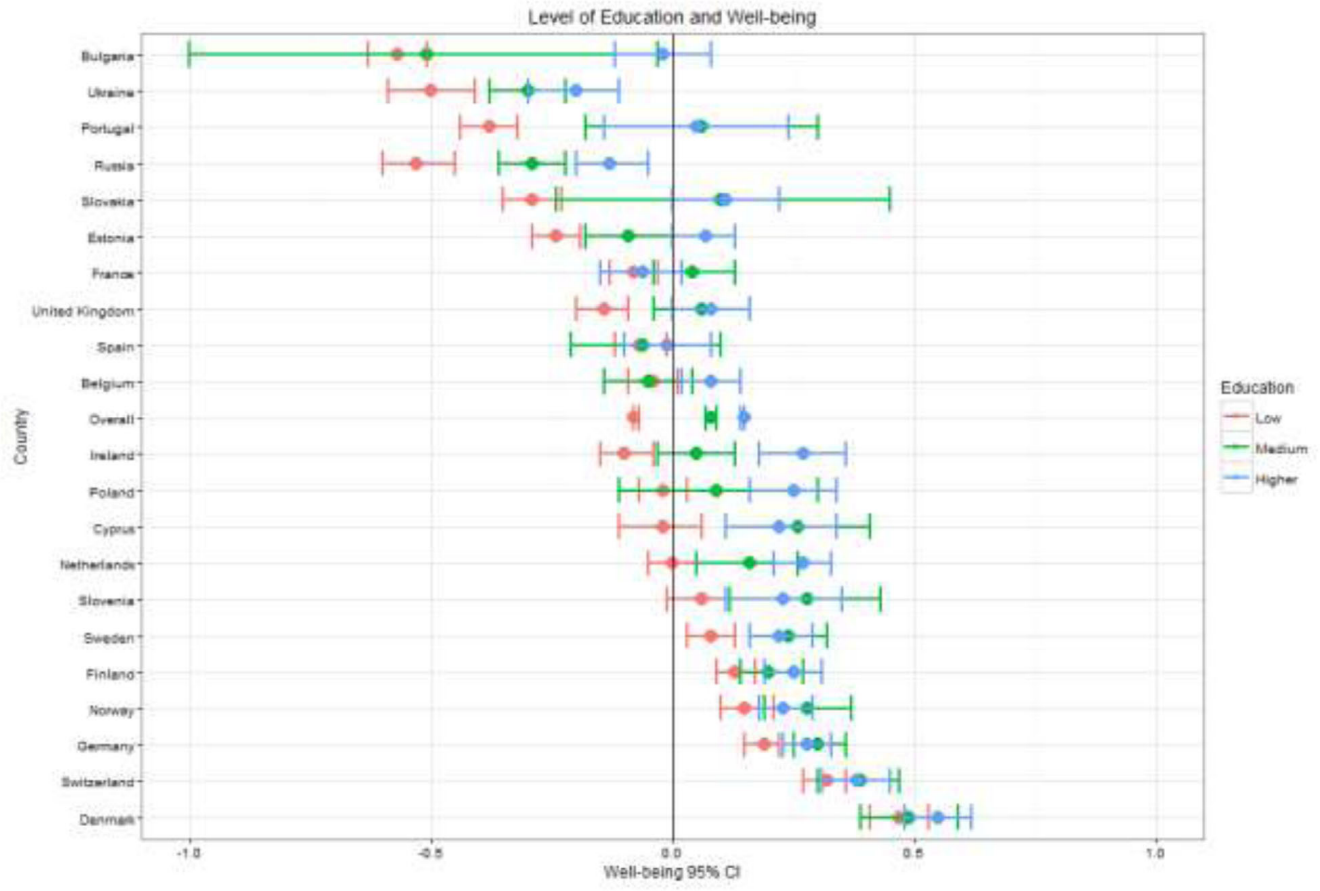
Well-being by country and education

Denmark having the highest well-being is consistent with many studies [4, 18] and with previous work using ESS data [27]. While the pattern is typically that Nordic countries are doing the best and that eastern countries have the lowest well-being, exceptions exist. The most notable exception is Portugal, which has the third-lowest score and is not significantly higher than Ukraine, which is second lowest. Switzerland and Germany are second and third highest respectively, and show generally similar patterns to the Scandinavian countries (see Fig. 1). It should be noted that, for Figs. 1, 2, 3, 4, 5, countries with the lowest well-being are at the top. This is done to highlight the greatest areas for potential impact, which are also the most of concern to policy.

General patterns across the key demographic variables – gender, age, education, employment – are visible across countries as seen in Figs. 1, 2, 3, 4, 5 (see also Supplement 2). These figures highlight patterns based on overall well-being as well as potential for inequalities. The visualizations presented here, though univariate, are for the purpose of understanding broad patterns while highlighting the need to disentangle groups and specific dimensions to generate effective policies.

For gender, women exhibited lower MPWB scores than men across Europe (β = −.09, *t* (36508) = − 10.37; *p* < .001). However, these results must be interpreted with caution due to considerable overlap in confidence intervals for many of the countries, and greater exploration of related variables is required. This also applies for the five countries (Estonia, Finland, Ireland, Slovakia, Ukraine) where women have higher means than men. Only four countries have significant differences between genders, all of which involve men having higher scores than women: the Netherlands (β = −.12, *t* (1759) = − 3.24; *p* < .001), Belgium (β = −.14, *t* (1783) = − 3.94; *p* < .001), Cyprus (β = −.18, *t* (930) = − 2.87; *p* < .001) and Portugal (β = −.19, *t* (1847) = − 2.50; *p* < .001).

While older individuals typically exhibited lower MPWB scores compared to younger age groups across Europe (β_25–44_ = −.05, *t* (36506) = − 3.686, *p* < .001; β_45–65_ = −.12, *t* (36506) = − 8.356, *p* < .001; β_65–74_ = −.16, *t* (36506) = − 8.807, *p* < .001; β_75+_ = −.28, *t* (36506) = − 13.568, *p* < .001), the more compelling pattern shows more extreme differences within and between age groups for the six countries with the lowest well-being. This pattern is most pronounced in Bulgaria, which has the lowest overall well-being. For the three countries with the highest well-being (Denmark, Switzerland, Germany), even the mean of the oldest age group was well above the European average, while for the countries with the lowest well-being, it was only young people, particularly those under 25, who scored above the European average. With the exception of France and Denmark, countries with higher well-being typically had fewer age group differences and less variance within or between groups. Only countries with the lowest well-being showed age differences that were significant with those 75 and over showing the lowest well-being.

MPWB is consistently higher for employed individuals and students than for retired (β = −.31, *t* (36506) = − 21.785; *p* < .00) or unemployed individuals (β = −.52, *t* (36556) = − 28.972; *p* < .001). Unemployed groups were lowest in nearly all of the 21 countries, though the size of the distance from other groups did not consistently correlate with national MPWB mean. Unemployed individuals in the six countries with the lowest well-being were significantly below the mean, though there is little consistency across groups and countries by employment beyond that. In countries with high well-being, unemployed, and, in some cases, retired individuals, had means below the European average. In countries with the lowest well-being, it was almost exclusively students who scored above the European average. Means for retired groups appear to correlate most strongly with overall well-being. There is minimal variability for employed groups in MPWB means within and between countries.

There is a clear pattern of MPWB scores increasing with education level, though the differences were most pronounced between low and middle education groups (β = .12, *t* (36508) = 9.538; *p* < .001). Individuals with high education were significantly higher on MPWB than those in the middle education group (β = .10, *t* (36508) =11.06; *p* < .001). Differences between groups were noticeably larger for countries with lower overall well-being, and the difference was particularly striking in Bulgaria. In Portugal, medium and high education well-being means were above the European average (though 95% confidence intervals crossed 0), but educational attainment is significantly lower in the country, meaning the low education group represents a greater proportion of the population than the other 21 countries. In the six countries with the highest well-being, mean scores for all levels of education were above the European mean.

### Utilizing ten dimensions for superior understanding of well-being

**Fig. 6:**
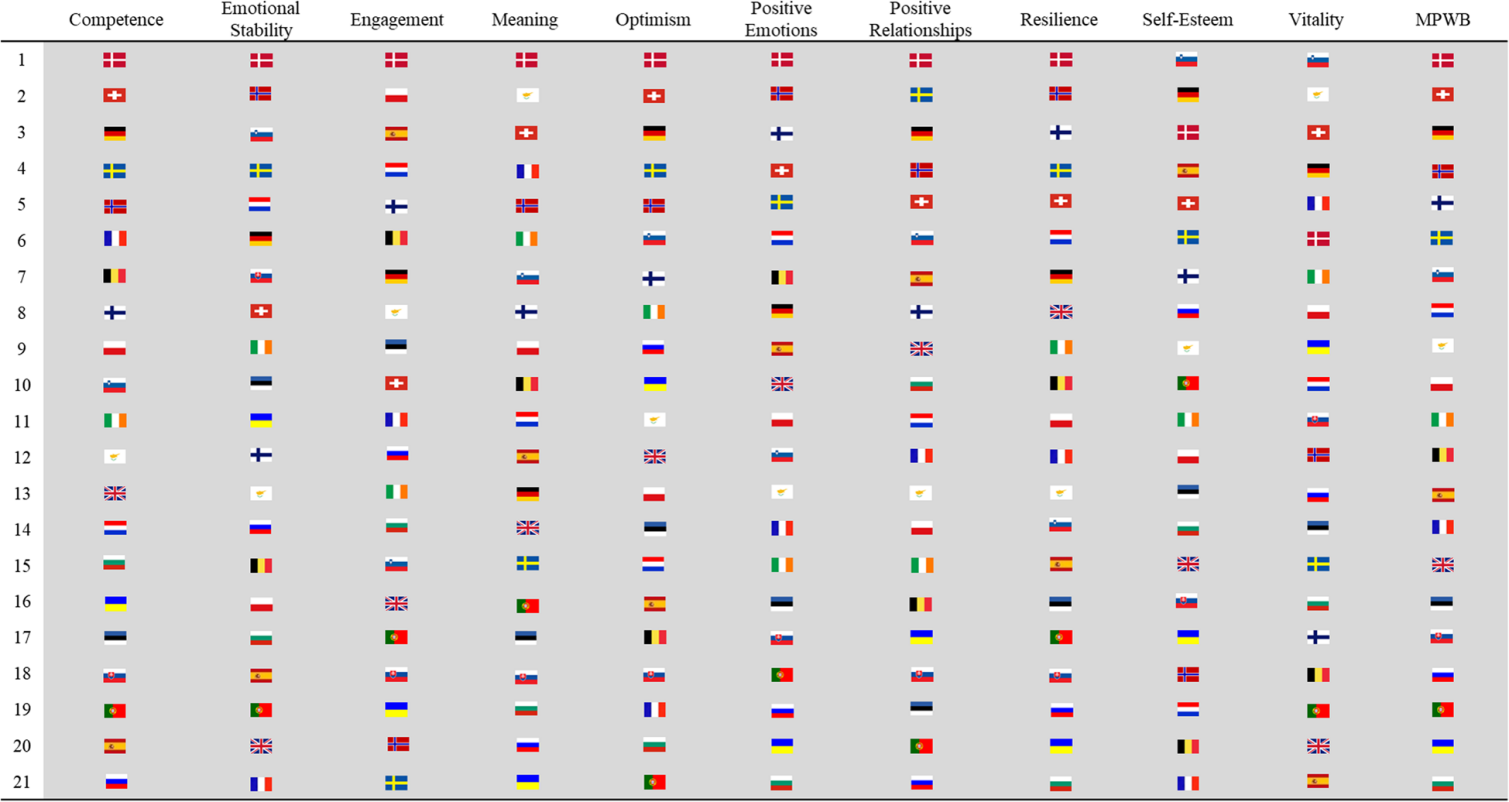
Country rankings in 2012 on multidimensional psychological well-being and each of its 10 dimensions

It is common to find rankings of national happiness and well-being in popular literature. Similarly, life satisfaction is routinely the only measure reported in many policy documents related to population well-being. To demonstrate why such limited descriptive approaches can be problematic, and better understood using multiple dimensions, all 21 countries were ranked individually on each of the 10 indicators of well-being and MPWB in Round 6 based on their means. Figure 6 demonstrates the variations in ranking across the 10 dimensions of well-being for each country.

**Fig. 7:**
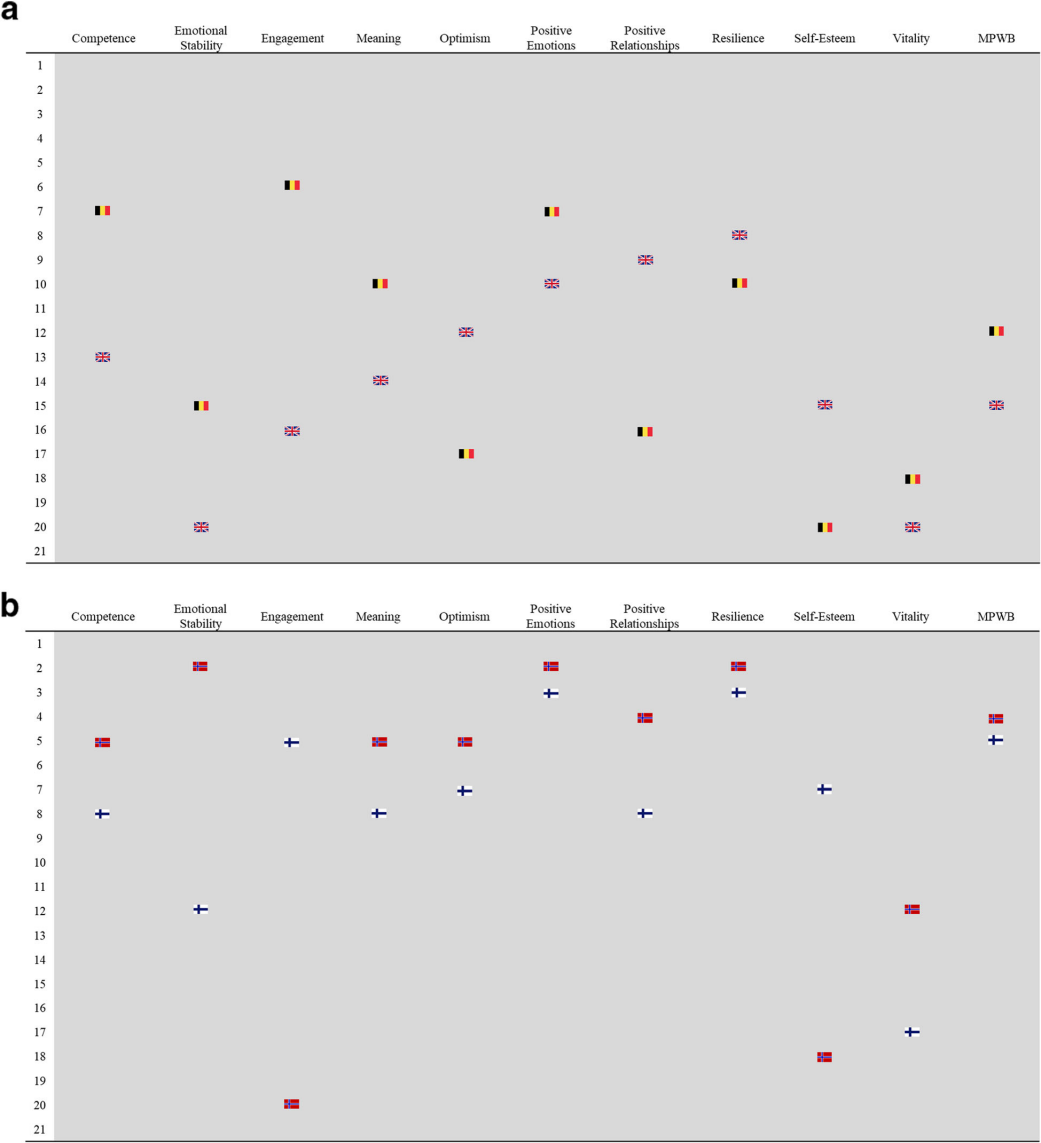
**a** Comparison of ranks for dimensions of well-being between two different countries with similar life satisfaction in 2012: Belgium and United Kingdom. **b** Comparison of ranks for dimensions of well-being between two similar countries with identical life satisfaction and composite well-being scores in 2012: Finland and Norway

The general pattern shows typically higher rankings for well-being dimensions in countries with higher overall well-being (and vice-versa). Yet countries can have very similar scores on the composite measure but very different underlying profiles in terms of individual dimensions. Figure 7a presents this for two countries with similar life satisfaction and composite well-being, Belgium and the United Kingdom. Figure 7b then demonstrates this even more vividly for two countries, Finland and Norway, which have similar composite well-being scores and identical mean life satisfaction scores (8.1), as well as have the highest two values for happiness of all 21 countries. In both pairings, the broad outcomes are similar, yet countries consistently have very different underlying profiles in individual dimensions. The results indicate that while overall scores can be useful for general assessment, specific dimensions may vary substantially, which is a relevant first step for developing interventions. Whereas the ten items are individual measures of 10 areas of well-being, had these been limited to a single domain only, the richness of the underlying patterns would have been lost, and the limitation of single item approaches amplified.

## Discussion

The ten-item multidimensional measure provided clear patterns for well-being across 21 countries and various groups within. Whether used individually or combined into a composite score, this approach produces more insight into well-being and its components than a single item measure such as happiness or life satisfaction. Fundamentally, single items are impossible to unpack in reverse to gain insights, whereas the composite score can be used as a macro-indicator for more efficient overviews as well as deconstructed to look for strengths and weaknesses within a population, as depicted in Figs. 6 and 7. Such deconstruction makes it possible to more appropriately target interventions. This brings measurement of well-being in policy contexts in line with approaches like GDP or national ageing indexes [7], which are composite indicators of many critical dimensions. The comparison with GDP is discussed at length in the following sections.

### Patterns within and between populations

Overall, the patterns and profiles presented indicate a number of general and more nuanced insights. The most consistent among these is that the general trend in national well-being is usually matched within each of the primary indicators assessed, such as lower well-being within unemployed groups in countries with lower overall scores than in those with higher overall scores. While there are certainly exceptions, this general pattern is visible across most indicators.

The other general trend is that groups with lower MPWB scores consistently demonstrate greater variability and wider confidence intervals than groups with higher scores. This is a particularly relevant message for policymakers given that it is an indication of the complexity of inequalities: improvements for those doing well may be more similar in nature than for those doing poorly. This is particularly true for employment versus unemployment, yet reversed for educational attainment. Within each dimension, the most critical pattern is the lack of consistency for how each country ranks, as discussed further in other sections.

Examining individual dimensions of well-being makes it possible to develop a more nuanced understanding of how well-being is impacted by societal indicators, such as inequality or education. For example, it is possible that spending more money on education improves well-being on some dimensions but not others. Such an understanding is crucial for the implementation of targeted policy interventions that aim at weaker dimensions of well-being and may help avoid the development of ineffective policy programs. It is also important to note that the patterns across sociodemographic variables may differ when all groups are combined, compared to results within countries. Some effects may be larger when all are combined, whereas others may have cancelling effects.

Using these insights, one group that may be particularly important to consider is unemployed adults, who consistently have lower well-being than employed individuals. Previous research on unemployment and well-being has often focused on mental health problems among the unemployed [46] but there are also numerous studies of differences in positive aspects of well-being, mainly life satisfaction and happiness [22]. A large population-based study has demonstrated that unemployment is more strongly associated with the absence of positive well-being than with the presence of symptoms of psychological distress [28], suggesting that programs that aim to increase well-being among unemployed people may be more effective than programs that seek to reduce psychological distress.

Certainly, it is well known that higher income is related to higher subjective well-being and better health and life expectancy [1, 42], so reduced income following unemployment is likely to lead to increased inequalities. Further work would be particularly insightful if it included links to specific dimensions of well-being, not only the comprehensive scores or overall life satisfaction for unemployed populations. As such, effective responses would involve implementation of interventions known to increase well-being in these groups in times of (or in spite of) low access to work, targeting dimensions most responsible for low overall well-being. Further work on this subject will be presented in forthcoming papers with extended use of these data.

This thinking also applies to older and retired populations in highly deprived regions where access to social services and pensions are limited. A key example of this is the absence in our data of a U-shaped curve for age, which is commonly found in studies using life satisfaction or happiness [5]. In our results, older individuals are typically lower than what would be expected in a U distribution, and in some cases, the oldest populations have the lowest MPWB scores. While previous studies have shown some decline in well-being beyond the age of 75 [20], our analysis demonstrates quite a severe fall in MPWB in most countries. What makes this insight useful – as opposed to merely unexpected – is the inclusion of the individual dimensions such as vitality and positive relationships. These dimensions are clearly much more likely to elicit lower scores than for younger age groups. For example, ageing beyond 75 is often associated with increased loneliness and isolation [33, 43], and reduction in safe, independent mobility [31], which may therefore correspond with lower scores on positive relationships, engagement, and vitality, and ultimately lower scores on MPWB than younger populations. Unpacking the dimensions associated with the age-related decline in well-being should be the subject of future research. The moderate positive relationship of MPWB scores with life satisfaction is clear but also not absolute, indicating greater insights through multidimensional approaches without any obvious loss of information. Based on the findings presented here, it is clearly important to consider ensuring the well-being of such groups, the most vulnerable in society, during periods of major social spending limitations.

### Policy implications

Critically, Fig. 6 represents the diversity of how countries reach an overall MPWB score. While countries with overall high well-being have typically higher ranks on individual items, there are clearly weak dimensions for individual countries. Conversely, even countries with overall low well-being have positive scores on some dimensions. As such, the lower items can be seen as potential policy levers in terms of targeting areas of concern through evidence-based interventions that should improve them. Similarly, stronger areas can be seen as learning opportunities to understand what may be driving results, and thus used to both sustain those levels as well as potentially to translate for individuals or groups not performing as well in that dimension. Collectively, we can view this insight as a message about specific areas to target for improvement, even in countries doing well, and that even countries doing poorly may offer strengths that can be enhanced or maintained, and could be further studied for potential applications to address deficits. We sound a note of caution however, in that these patterns are based on ranks rather than actual values, and that those ranks are based on single measures.

Figure 7 complements those insights more specifically by showing how Finland and Norway, with a number of social, demographic, and economic similarities, plus identical life satisfaction scores (8.1) arrive at similar single MPWB scores with very different profiles for individual dimensions. By understanding the levers that are specific to each country (i.e. dimensions with the lowest well-being scores), policymakers can respond with appropriate interventions, thereby maximizing the potential for impact on entire populations. Had we restricted well-being measurement to a single question about happiness, as is commonly done, we would have seen both countries had similar and extremely high means for happiness. This might have led to the conclusion that there was minimal need for interventions for improving well-being. Thus, in isolation, using happiness as the single indicator would have masked the considerable variability on several other dimensions, especially those dimensions where one or both had means among the lowest of the 21 countries. This would have resulted in similar policy recommendations, when in fact, Norway may have been best served by, for example, targeting lower dimensions such as Engagement and Self-Esteem, and Finland best served by targeting Vitality and Emotional Stability.

Targeting specific groups and relevant dimensions as opposed to comparing overall national outcomes between countries is perhaps best exemplified by Portugal, which has one of the lowest educational attainment rates in OECD countries, exceeded only by Mexico and Turkey [36]. This group thus skews the national MPWB score, which is above average for middle and high education groups, but much lower for those with low education. Though this pattern is not atypical for the 21 countries presented here, the size of the low education group proportional to Portugal’s population clearly reduces the national MPWB score. This implies that the greatest potential for improvement is likely to be through addressing the well-being of those with low education as a near-term strategy, and improving access to education as a longer-term strategy. It will be important to analyze this in the near future, given recent reports that educational attainment in Portugal has increased considerably in recent years (though remains one of the lowest in OECD countries) [36].

One topic that could not be addressed directly is whether these measures offer value as indicators of well-being beyond the 21 countries included here, or even beyond the countries included in ESS generally. In other words, are these measures relevant only to a European population or is our approach to well-being measurement translatable to other regions and purposes? Broadly speaking, the development of these measures being based on DSM and ICD criteria should make them relevant beyond just the 21 countries, as those systems are generally intended to be global. However, it can certainly be argued that these methods for designing measures are heavily influenced by North American and European medical frameworks, which may limit their appropriateness if applied in other regions. Further research on these measures should consider this by adding potential further measures deemed culturally appropriate and seeing if comparable models appear as a result.

### A single well-being score

One potential weakness remains the inconsistency of scaling between ESS well-being items used for calculating MPWB. However, this also presents an opportunity to consider the relative weighting of each item within the current scales, and allow for the development of a more consistent and reliable measure. These scales could be modified to align in separate studies with new weights generated – either generically for all populations or stratified to account for various cultural or other influences. Using these insights, scales could alternatively be produced to allow for simple scoring for a more universally accessible structure (e.g. 1–100) but with appropriate values for each item that represents the dimensions, if this results in more effective communication with a general public than a standardized score with weights. Additionally, common scales would improve on attempts to use rankings for presenting national variability within and between dimensions. Researchers should be aware that factor scores are sample-dependent (as based on specific factor model parameters such as factor loadings). Nevertheless, future research focused on investigating specific item differential functioning (by means of multidimensional item response functioning or akin techniques) of these items across situations (i.e., rounds) and samples (i.e., rounds and countries) should be conducted in order to have a more nuanced understanding of this scale functioning.

What makes this discussion highly relevant is the value of a more informed measure to replace traditional indicators of well-being, predominantly life satisfaction. While life satisfaction may have an extensive history and present a useful metric for comparisons between major populations of interest, it is at best a corollary, or natural consequence, of other indicators. It is not in itself useful for informing interventions, in the same way limiting to a single item for any specific dimension of well-being should not alone inform interventions.

By contrast, a validated and standardized multidimensional measure is exceptionally useful in its suitability to identify those at risk, as well as its potential for identifying areas of strengths and weaknesses within the at-risk population. This can considerably improve the efficiency and appropriateness of interventions. It identifies well-understood dimensions (e.g. vitality, positive emotion) for direct application of evidence-based approaches that would improve areas of concern and thus overall well-being. Given these points, we strongly argue for the use of multidimensional approaches to measurement of well-being for setting local and national policy agenda.

There are other existing single-score approaches for well-being addressing its multidimensional nature. These include the Warwick-Edinburgh Mental Well-Being Scale [44] and the Flourishing Scale [11]. In these measures, although the single score is derived from items that clearly tap a number of dimensions, the dimensions have not been systematically derived and no attempt is made to measure the underlying dimensions individually. In contrast, the development approach used here – taking established dimensions from DSM and ICD – is based on years of international expertise in the field of mental illness. In other words, there have long been adequate measures for identifying and understanding illness, but there is room for improvement to better identify and understand health. With increasing support for the idea of these being a more central focus of primary outcomes within economic policies, such approaches are exceptionally useful [13].

### Better measures, better insights

Naturally, it is not a compelling argument to simply state that more measures present greater information than fewer or single measures, and this is not the primary argument of this manuscript. In many instances, national measures of well-being are mandated to be restricted to a limited set of items. What is instead being argued is that well-being itself is a multidimensional construct, and if it is deemed a critical insight for establishing policy agenda or evaluating outcomes, measurements must follow suit and not treat happiness and life satisfaction values as universally indicative. The items included in ESS present a very useful step to that end, even in a context where the number of items is limited.

As has been argued by many, greater consistency in measurement of well-being is also needed [26]. This may come in the form of more consistency regarding dimensions included, the way items are scored, the number of items representing each dimension, and changes in items over time. While inconsistency may be prevalent in the literature to date for definitions and measurement, the significant number of converging findings indicates increasingly robust insights for well-being relevant to scientists and policymakers. Improvements to this end would support more systematic study of (and interventions for) population well-being, even in cases where data collection may be limited to a small number of items.

### The added value of MPWB as a composite measure

While there are many published arguments (which we echo) that measures of well-being must go beyond objective features, particularly related to economic indicators such as GDP, this is not to say one replaces the other. More practically, subjective and objective approaches will covary to some degree but remain largely distinct. For example, GDP presents a useful composite of a substantial number of dimensions, such as consumption, imports, exports, specific market outcomes, and incomes. If measurement is restricted to a macro-level indicator such as GDP, we cannot be confident in selecting appropriate policies to implement. Policies are most effective when they target a specific component (of GDP, in this instance), and then are directly evaluated in terms of changes in that component. The composite can then be useful for comprehensive understanding of change over time and variation in circumstances. Specific dimensions are necessary for identifying strengths and weaknesses to guide policy, and examining direct impacts on those dimensions. In this way, a composite measure in the form of MPWB for aggregate well-being is also useful, so long as the individual dimensions are used in the development and evaluation of policies. Similar arguments for other multidimensional constructs have been made recently, such as national indexes of ageing [7].

In the specific instance of MPWB in relation to existing measures of well-being, there are several critical reasons to ensure a robust approach to measurement through systematic validation of psychometric properties. The first is that these measures are already part of the ESS, meaning they are being used to study a very large sample across a number of social challenges and not specifically a *new* measure for well-being. The ESS has a significant influence on policy discussions, which means the best approaches to utilizing the data are critical to present systematically, as we have attempted to do here. This approach goes beyond existing measures such as Gallup or the World Happiness Index to broadly cover psychological well-being, not individual features such as happiness or life satisfaction (though we reiterate: as we demonstrate in Fig. 7a and b, these individual measures can and should still covary broadly with any multidimensional measure of well-being, even if not useful for predicting all dimensions). While often referred to as ‘comprehensive’ measurement, this merely describes a broad range of dimensions, though more items for each dimension – and potentially more dimensions – would certainly be preferable in an ideal scenario.

These dimensions were identified following extensive study for flourishing measures by Huppert & So [27], meaning they are not simply a mix of dimensions, but established systematically as the key features of well-being (the opposite of ill-being). Furthermore, the development of the items is in line with widely validated and practiced measures for the identification of illness. The primary adjustment has simply been the emphasis on health, but otherwise maintains the same principles of assessment. Therefore, the overall approach offers greater value than assessing only negative features and inferring absence equates to opposite (positives), or that individual measures such as happiness can sufficiently represent a multidimensional construct like well-being. Collectively, we feel the approach presented in this work is therefore a preferable method for assessing well-being, particularly on a population level, and similar approaches should replace single items used in isolation.

## Conclusions

While the focus of this paper is on the utilization of a widely tested measure (in terms of geographic spread) that provides for assessing population well-being, it is important to provide a specific application for why this is relevant in a policy context. Additionally, because the ESS itself is a widely-recognized source of meaningful information for policymakers, providing a robust and comprehensive exploration of the data is necessary. As the well-being module was not collected in recent rounds, these insights provide clear reasoning and applications for bringing them back in the near future.

More specifically, it is critical that this approach be seen as advantageous both in using the composite measure for identifying major patterns within and between populations, and for systematically unpacking individual dimensions. Using those dimensions produces nuanced insights as well as the possibility of illuminating policy priorities for intervention.

In line with this, we argue that no composite measure can be useful for developing, implementing, or evaluating policy if individual dimensions are not disaggregated. We are not arguing that MPWB as a single composite score, nor the additional measures used in ESS, is better than other existing single composite scoring measures of well-being. Our primary argument is instead that MPWB is constructed and analyzed specifically for the purpose of having a robust measure suitable for disaggregating critical dimensions of well-being. Without such disaggregation, single composite measures are of limited use. In other words, construct a composite and target the components.

Well-being is perhaps the most critical outcome measure of policies. Each individual dimension of well-being as measured in this study represents a component linked to important areas of life, such as physical health, financial choice, and academic performance [26]. For such significant datasets as the European Social Survey, the use of the single score based on the ten dimensions included in multidimensional psychological well-being gives the ability to present national patterns and major demographic categories as well as to explore specific dimensions within specific groups. This offers a robust approach for policy purposes, on both macro and micro levels. This facilitates the implementation and evaluation of interventions aimed at directly improving outcomes in terms of population well-being.

## Supplementary information

**Additional file 1: Figure S1**. Hierarchical approach to modelling comprehensive psychological well-being. **Table S1**. Confirmatory Factor Structure for Round 6 and 3. **Figure S2**. Well-being by country and gender. **Figure S3**. Well-being by country and age. **Figure S4**. Well-being by country and employment. **Figure S5**. Well-being by country and education. **Table S2**. Item loadings for Belgium to Great Britain. **Table S3**. Item loadings for Ireland to Ukraine.

## Data Availability

The datasets analysed during the current study are available in the European Social Survey repository, http://www.europeansocialsurvey.org/data/country_index.html

## Abbreviations

DSM: Diagnostic and Statistical Manual of Mental Disorders
ESS: European Social Survey
GDP: Gross Domestic Product
ICD: International Classification of Disease
MPWB: Multidimensional psychological well-being
