# Supplementary material for: Well-being is more than happiness and life satisfaction: a multidimensional analysis of 21 countries
Source: Health Qual Life Outcomes. 2020 Jun 19;18:192. doi: 10.1186/s12955-020-01423-y (PMC7304199; doi:10.1186/s12955-020-01423-y)
Supplement: Supplementary file 1 — Additional file 1: Figure S1. Hierarchical approach to modelling comprehensive psychological well-being. Table S1. Confirmatory Factor Structure for Round 6 and 3. Figure S2. Well-being by country and gender. Figure S3. Well-being by country and age. Figure S4. Well-being by country and employment. Figure S5. Well-being by country and education. Table S2. Item loadings for Belgium to Great Britain. Table S3. Item loadings for Ireland to Ukraine. [file 12955_2020_1423_MOESM1_ESM.docx]

**Supplementary Materials**

**Well-being is more than happiness and life satisfaction: A multidimensional analysis of 21 countries**

**Analyses involving comprehensive psychological well-being**

For the purposes of this paper, the key analyses focused on major social indicators, namely patterns in well-being between countries and by age, gender, education, and employment. Additionally, macro-level inequalities are tested for the same variables within the same analyses as well as an overall inequality analysis. The purpose of this paper was to present a robust method for calculating well-being across Europe using the ESS data. Analysis of change between years will be reported separately. The inclusion of both Round 3 and Round 6 in this paper is for the purpose of showing the method is appropriate for building well-being indicators using the available data for both rounds as well as with any future iterations of the ESS. Results primarily focus on the Round 6 data as Round 3 insights have been widely published given they have been available since 2007. The only exception to this has been provided purely as a case example for applications in a policy context.


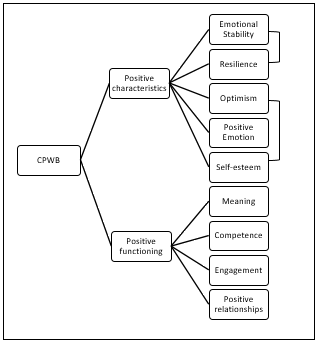


*Figure S1*. Hierarchical approach to modelling comprehensive psychological well-being.

**CPWB factor structure differences across groups**

To provide a descriptive evaluation of factor analysis parameters across different groups, factor parameters are presented for round 3 and round 6, for which the CPWB scores are later obtained. In this sense, the parameter estimates are similar in both rounds (Table S1). The biggest difference is found for the Engagement item, which was expected to occur given that the item was changed across rounds. It could be observed that the positive relationship item presents the lowest loading on its substantive factor. Additionally, the correlations between the two pairs of items with similar wording (i.e. Emotional Stability and Vitality items and Optimism and Self Esteem items) was stable and similar across rounds.

Table S1.Confirmatory Factor Structure for Round 6 and 3.

|  | Round 6 | | | Round 3 | | |
| --- | --- | --- | --- | --- | --- | --- |
| Item | CPWB | PC | PF | CPWB | PC | PF |
| Emotional Stability |  | .49 |  |  | .46 |  |
| Vitality |  | .49 |  |  | .50 |  |
| Resilience |  | .45 |  |  | .42 |  |
| Optimism |  | .56 |  |  | .59 |  |
| Positive emotion |  | .59 |  |  | .57 |  |
| Self-esteem |  | .52 |  |  | .54 |  |
| Engagement* |  |  | .48 |  |  | .41 |
| Meaning |  |  | .57 |  |  | .59 |
| Positive relationships |  |  | .35 |  |  | .36 |
| Competence* |  |  | .64 |  |  | .65 |
| Positive Characteristics | .92 |  |  | .88 |  |  |
| Positive Characteristics | .92 |  |  | .88 |  |  |
|  |  |  |  |  |  |  |
| Item residual correlations |  |  |  |  |  |  |
| Emotional Stability – Vitality | .21 |  |  | .20 |  |  |
| Optimism – Self-steem | .29 |  |  | .26 |  |  |
| *Note:* CPWB: Comprehensive Well-being factor; PC = Positive Characteristics; PF = Positive Functioning. * Item changed across rounds. | | | | | | |

**CPWB convergent validity**

CPWB scores were expected to inversely predict the ill-being constructs (i.e., depression) and to be positively related with alternative measures of well-being. The wellbeing module in Rounds 3 and 6 included several measures for testing such relationships: Depression was assessed by the Center for Epidemiologic Studies – Depression Scale (CES-D 8; Radloff, 1977), which was included in both, round 3 and 6. Alternative measures of well-being encompassed the Five Ways to well-being (which covers five well-being related activities such as connect, be active, take notice, learn and give; New Economics Foundation, 2008), which is included in round 6.

CES-D 8 consists of 8 items measured in a Likert-type scale ranging from 0 (e.g., “I feel depressed none of the time” to 3 (e.g. “I feel depressed all the time”). In addition, the Five Ways to well-being are measured with 5 items ranging from 0 to 6 (Learn, Connect), 0-7 (Be active), 1-7 (Give) and 0-10 (Take notice). A model including CPWB as a predictor of a general factor of depression (estimated from CES-D items) and the Five ways of well-being (only round 6) was computed. As predicted, CPWB was negatively related to depression (round 3: β = -.878, p <.001; round 6: β = -.760, p <.001) and positively predicted all 5 ways to well-being: learn (β = .155, p <.001), take notice (β = .139, p <.001) connect (β = .247, p <.001), connect (β = .024, p <.001) and physical activity (β = .017, p <.001).

**CPWB measurement invariance**

The establishment of measurement invariance was a challenge due to several considerations: (a) As two items were changed across rounds, we were unable to perform measurement analysis, as the parameters involving such items would always differ. (b) Even if the items were comparable across rounds, the high sample size makes impossible the use of traditional measurement invariance techniques, such as multigroup confirmatory factor analysis (i.e. to search for strict measurement variance comparing configural, scalar and metric models across groups); (c)these techniques are unreliable when testing a high number of comparisons, as in our case.

To overcome these challenges several alternatives were available, such as multilevel CFA, multilevel factor mixture modelling, Bayesian approximate invariance models,alignment optimization (Kim, Cao, Wang & Nguyen, 2017) or Expected Parameter Change (EPC; Oberski, 2017). However the benefits and drawback of each specific method are yet to be established. Given that our models of interest involve a high number of possible sources of invariance, early inspections of scalar invariance using different approximate measurement invariances were unable to establish such invariance across groups within a single round. As acknowledged in the literature, even these modern techniques fail to provide substantive answers if a large number of potential sources of model invariance are present (Oberski, 2017), as occurs in our case. Similar concerns have been raised with other widely accepted measures in the literature (i.e. GDP; Hosseiny, 2016).

In light of these results, we decided to use factor scores for computing our CPWB scores. It is acknowledged that factor scores bring challenges of its own (i.e., factor indeterminacy), but its use allows the comparison of CPWB across countries and groups. Factor indeterminacy arises from the factors not being unique with respect to the variables included in the model (Beauducel & Hilger, 2017). This leads to infinite sets of factor scores being derived from a common factor model. Yet, if the scores are determinate enough, the ranking of individuals in the all potential sets of factor scores would yield similar results (Mulaik, 2010).

The use of factor scores is common in clinical (Kawashima & Shiomi, 2007) and cognitive (Bell, McCallum & Cox, 2003) settings, and an increasing area of research (Devlieger & Rosseel, 2017; Devlieger, Mayer & Rossel, 2015). Additionally, an approach similar to our CPWB scoring was applied in Grudke, et al., (2017) to study the differences in skill distribution across countries and industries using OECD data. In their study, the authors first apply exploratory factor analysis and derive factor scores – using regression methods – after comparing that the factor scores are reproduced across units of interest (e.g., countries). While our approach differs from Grudke et al., (2017) in several ways (i.e., use of confirmatory approach, different scoring method, logarithmic transformation of factor scores, etc.) the rationale of the two studies is comparable, and offers strong support for the analytical choices presented in this article. Other OECD articles (e.g., Nicoletti, Scarpetta & Boylaud, 2000) follow similar, but less refined, approaches to using factor analysis and factor weights for constructing composite scores.

Specifically, CPWB scores were computed using empirical Bayes a posteriori, which present better statistical properties than alternative methods (Eastbrook, 2013), using the lavaan package. Exact formulae can be found in Appendix 11 of Muthén (2004).

**References**

Bell, S. M., McCallum, R. S., & Cox, E. A. (2003). Toward a research-based assessment of dyslexia: Using cognitive measures to identify reading disabilities. *Journal of Learning Disabilities, 36*(6), 505-516.

Beauducel, A., & Hilger, N. (2017). On the Bias of Factor Score Determinacy Coefficients Based on Different Estimation Methods of the Exploratory Factor Model. Communications in Statistics – Simulation and computation, 46 (8), 6144-6154, DOI: [10.1080/03610918.2016.1197247](https://doi.org/10.1080/03610918.2016.1197247)

Devlieger, I., Mayer, A. & Rossel, Y. (2015). Hypothesis Testing Using Factor Score Regression: A Comparison of Fourth Methods. *Educational and Psychoogical Measurement, 76* (5), 741-770. DOI: 10.1177/0013164415607618

Devlieger, I & Rossel, Y. (2017). Factor Score Path Analysis. *Methodology, 13* (Supplement), 31-38. DOI: 10.1027/1614-2241/a000130

Eun Sook Kim, Chunhua Cao, Yan Wang & Diep T. Nguyen (2017) Measurement Invariance Testing with Many Groups: A Comparison of Five Approaches, *Structural Equation Modeling: A Multidisciplinary Journal (24)* 4, 524-544, DOI: 10.1080/10705511.2017.1304822

Grundke, R. et al. (2017), “Skills and global value chains: A characterisation”, OECD Science, Technology and Industry Working Papers, 2017/05, OECD Publishing, Paris. http://dx.doi.org/10.1787/cdb5de9b-en

Kawashima, N., & Shiomi, K. (2007). Factors of the thinking disposition of Japanese high school students. *Social Behavior and Personality, 35*(2), 187-194.

Hosseiny, A. (2015). Violation of Invariance of Measurement for GDP Growth Rate and Its Consequences*. ArXiv*. Retrieved from <https://arxiv.org/abs/1507.04848>.

Mulaik, S.A. (2010). *Foundations of factor analysis* (2nd Ed.). New York: CRC Press.

Muthén, B.O. (1998-2004*). Mplus Technical Appendices*. Los Angeles, CA: Muthén & Muthén.

Nicoletti, G., Scarpetta. S. & Boylaud. O. (2000). Summary Indicators of Product Market Regulation with an Extension to Employment Protection Legislation.

OECD Economics Department Working Papers, No. 226, OECD Publishing, Paris. http://dx.doi.org/10.1787/215182844604

Oberski, D. L. (2017). Sensitivity analysis for measurement invariance testing. In Davidov, E., Schmidt, P., Billiet, J., & Meuleman, B. (Eds.), *In Cross-cultural analysis: methods and applications, second edition.* Routledge.

Radloff, L.S. (1977). The CES-D Scale: A self-report depression scale for research in the general population. *Applied Psychological Measurement* (1). 385–401. doi: 10.1177/014662167700100306.

**Supplement 2 – Color versions of figures**

**Figure S2.** Well-being by country and gender

**
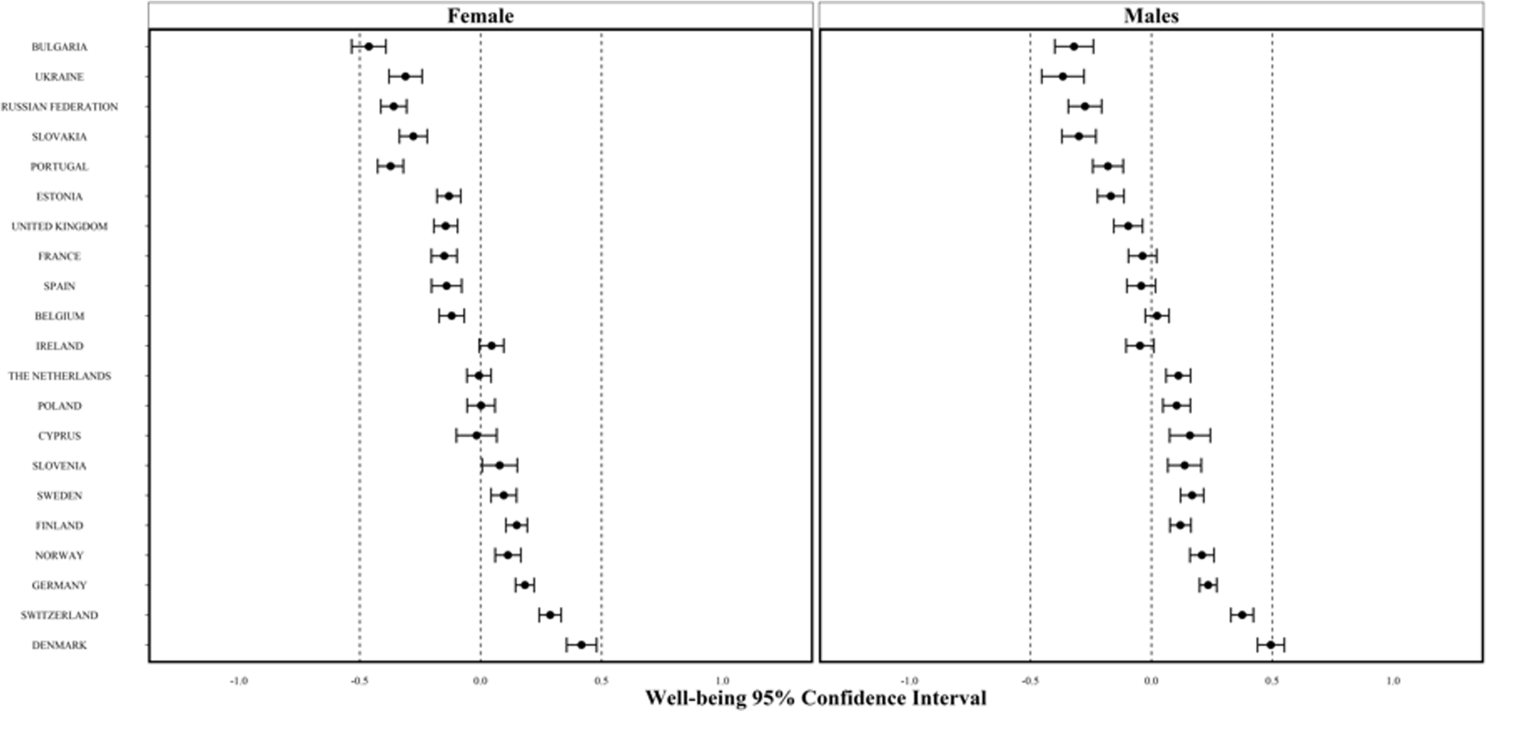
**

**Figure S3.** Well-being by country and age

**
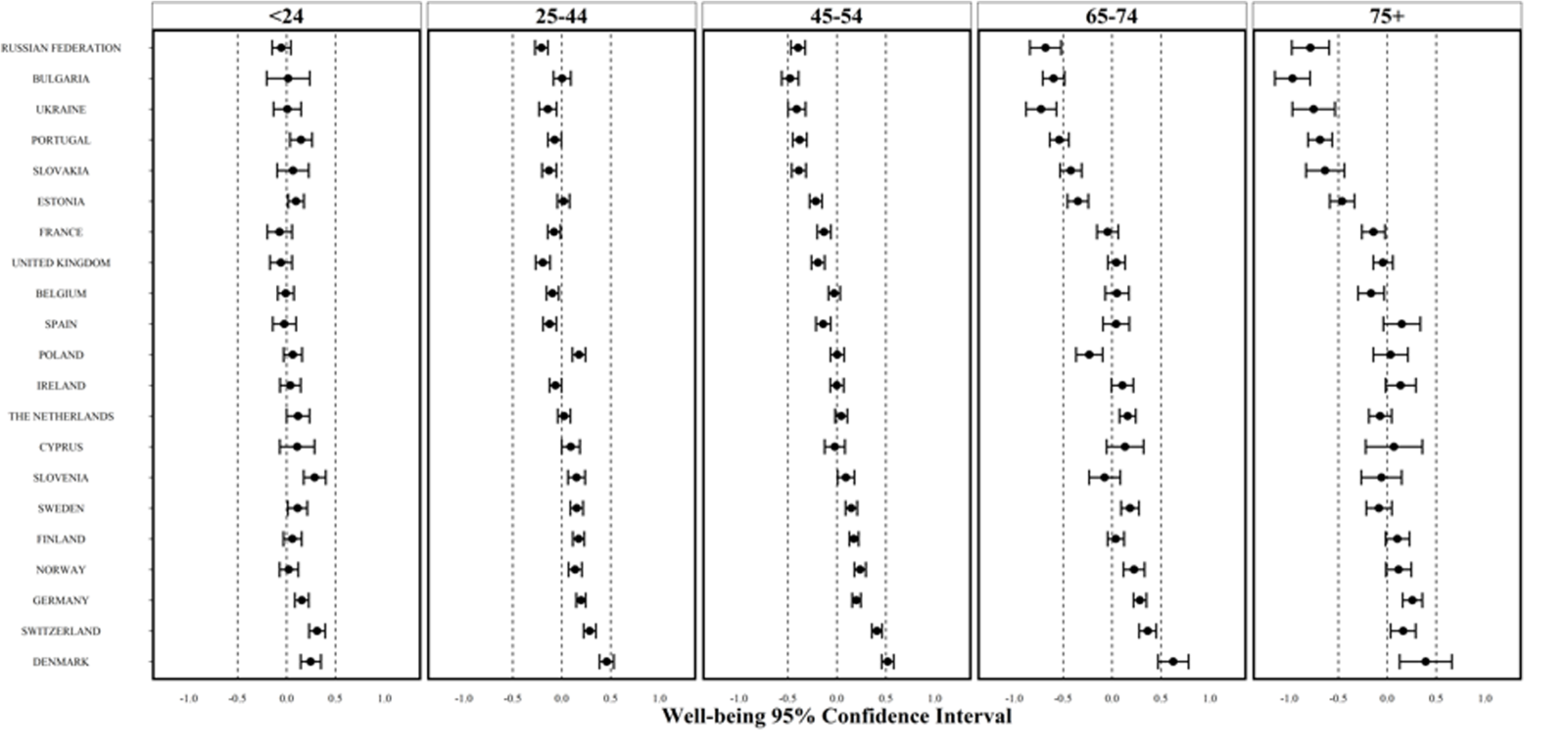
**

**Figure S4.** Well-being by country and employment

**
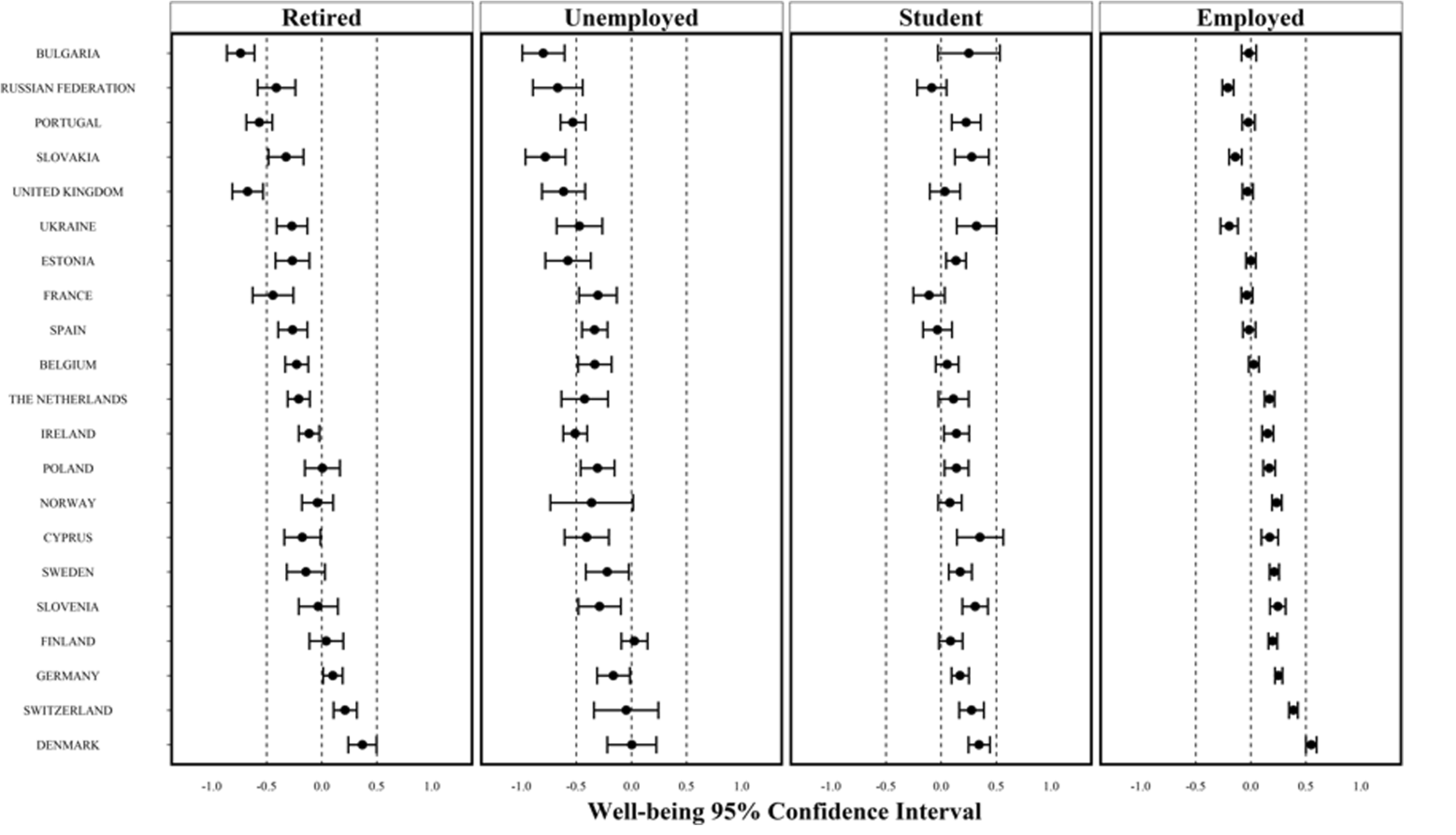
**

**Figure S5.** Well-being by country and education

**
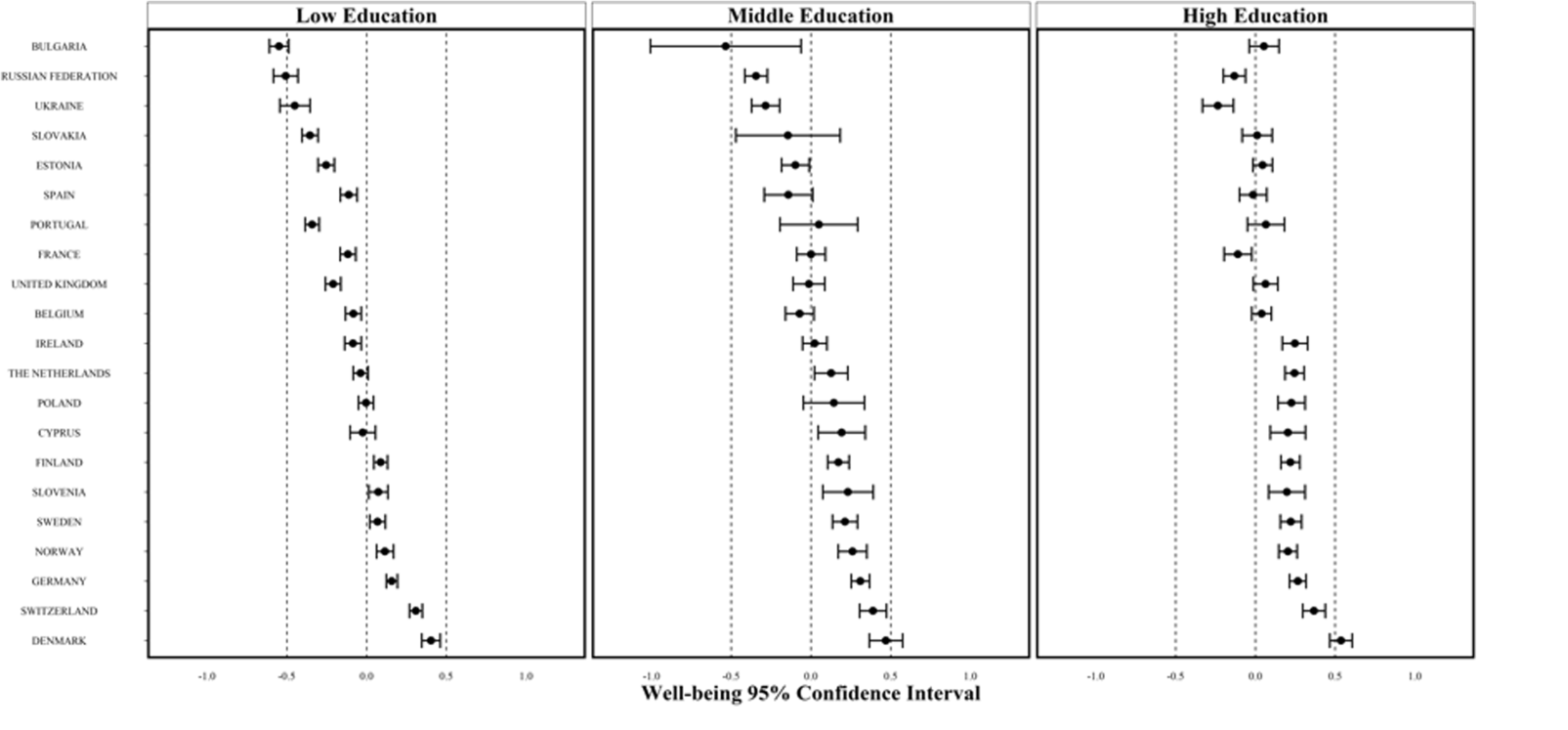
**

**Confirmatory Factor Analysis country by country**

Table S2: Item loadings for Belgium to Great Britain

|  | Belgium | Bulgaria | Switzerland | Cyprus | Germany | Denmark | Estonia | Spain | Finland | France | Great Britain |
| --- | --- | --- | --- | --- | --- | --- | --- | --- | --- | --- | --- |
| Emotional Stability | .426 | .635 | .501 | .515 | .432 | .524 | .440 | .540 | .451 | .509 | .517 |
| Vitality | .464 | .620 | .627 | .491 | .531 | .516 | .555 | .501 | .482 | .515 | .447 |
| Resilience | .465 | .480 | .407 | .412 | .445 | .560 | .420 | .465 | .382 | .434 | .500 |
| Optimism | .532 | .664 | .548 | .564 | .573 | .576 | .604 | .531 | .550 | .582 | .574 |
| Positive emotion | .574 | .713 | .564 | .618 | .551 | .621 | .685 | .651 | .644 | .472 | .648 |
| Self-esteem | .505 | .608 | .683 | .731 | .550 | .579 | .491 | .647 | .631 | .562 | .673 |
| Engagement* | .480 | .605 | .503 | .547 | .401 | .545 | .610 | .509 | .506 | .442 | .590 |
| Meaning | .601 | .681 | .524 | .639 | .481 | .467 | .600 | .400 | .604 | .570 | .620 |
| Positive relationships | .309 | .370 | .367 | .279 | .333 | .360 | .422 | .273 | .394 | .223 | .351 |
| Competence* | .625 | .763 | .605 | .628 | .555 | .560 | .518 | .608 | .553 | .618 | .664 |
| Positive Characteristics | .885 | .945 | .884 | .908 | .910 | .869 | .924 | .933 | .917 | .912 | .913 |
| Positive Functioning | .885 | .945 | .884 | .908 | .910 | .869 | .924 | .933 | .917 | .912 | .913 |
| Emotional Stability – Vitality | .076 | .287 | .213 | .384 | .146 | .210 | .312 | .185 | .162 | .142 | .175 |
| Optimism – Self-steem | .301 | .356 | .508 | .315 | .147 | .395 | .306 | .242 | .167 | .086 | .338 |
| **Model Fit** |  |  |  |  |  |  |  |  |  |  |  |
| N | 1787 | 1828 | 1428 | 933 | 2791 | 1311 | 2176 | 1498 | 2106 | 1853 | 2059 |
| χ^2^ | 116.667 | 17.294 | 61.382 | 86.664 | 162.931 | 82.045 | 182.783 | 114.543 | 168.818 | 187.007 | 155.654 |
| CFI | .985 | .994 | .990 | .990 | .984 | .992 | .987 | .989 | .984 | .977 | .991 |
| TLI | .980 | .992 | .986 | .986 | .978 | .988 | .982 | .984 | .977 | .967 | .987 |
| RMSEA | .028 | .030 | .020 | .030 | .028 | .023 | .032 | .029 | .032 | .037 | .028 |
|  | (.022- | (.026- | (.012- | (.022- | (.024- | (.017- | (.027- | (.024- | (.027- | (.032- | (.024- |
|  | .033) | .035) | .027) | .037) | .032) | .030) | .036) | .035) | .037) | .043) | .033) |
| SRMR | .027 | .027 | .026 | .029 | .028 | .029 | .029 | .028 | .033 | .035 | .027 |

Table S3: Item loadings for Ireland to Ukraine

|  | Ireland | The Netherlands | Norway | Poland | Portugal | Russian Federation | Sweden | Slovenia | Slovakia | Ukraine |
| --- | --- | --- | --- | --- | --- | --- | --- | --- | --- | --- |
| Emotional Stability | .545 | .504 | .467 | .385 | .534 | .459 | .483 | .455 | .597 | .418 |
| Vitality | .524 | .543 | .482 | .572 | .620 | .513 | .571 | .559 | .586 | .410 |
| Resilience | .545 | .526 | .486 | .386 | .343 | .384 | .495 | .442 | .435 | .300 |
| Optimism | .584 | .587 | .599 | .550 | .586 | .613 | .669 | .615 | .579 | .638 |
| Positive emotion | .620 | .675 | .626 | .569 | .643 | .584 | .600 | .591 | .580 | .593 |
| Self-esteem | .682 | .515 | .543 | .610 | .502 | .543 | .575 | .557 | .570 | .535 |
| Engagement* | .676 | .489 | .473 | .534 | .612 | .562 | .393 | .610 | .487 | .575 |
| Meaning | .656 | .633 | .580 | .510 | .611 | .541 | .603 | .635 | .672 | .539 |
| Positive relationships | .436 | .320 | .352 | .332 | .376 | .397 | .423 | .325 | .565 | .487 |
| Competence* | .698 | .558 | .572 | .674 | .637 | .629 | .634 | .648 | .591 | .593 |
| Positive Characteristics | .940 | .893 | .902 | .879 | .935 | .953 | .953 | .958 | .926 | .951 |
| Positive Functioning | .940 | .893 | .902 | .879 | .935 | .953 | .953 | .958 | .926 | .951 |
| Emotional Stability – Vitality | .337 | .086 | .188 | .258 | .218 | .301 | .231 | .220 | .346 | .395 |
| Optimism – Self-steem | .363 | .270 | .342 | .392 | .195 | .254 | .117 | .308 | .427 | .382 |
| **Model Fit** |  |  |  |  |  |  |  |  |  |  |
| N | 2384 | 1762 | 1578 | 1641 | 1850 | 1748 | 1753 | 1121 | 1583 | 1321 |
| χ2 | 212.263 | 139.494 | 101.609 | 116.147 | 208.335 | 179.682 | 188.139 | 106.598 | 92.592 | 224.927 |
| CFI | .993 | .987 | .990 | .987 | .986 | .985 | .985 | .990 | .995 | .971 |
| TLI | .990 | .982 | .986 | .982 | .981 | .979 | .980 | .986 | .994 | .960 |
| RMSEA | .029 | .030 | .025 | .029 | .038 | .037 | .034 | .030 | .023 | .050 |
|  | (.032- | (.035- | (.031- | (.035- | (.043- | (.042- | (.039- | (.036- | (.029- | (.056- |
|  | .025) | .025) | .020) | .024) | .033) | .032) | .029) | .024) | .018) | .044) |
| SRMR | .026 | .031 | .029 | .028 | .031 | .031 | .033 | .029 | .023 | .042 |
